# Adenosine deaminase acting on RNA1 mutation in chilblain lupus treated with tofacitinib

**DOI:** 10.1016/j.jdcr.2026.03.053

**Published:** 2026-03-30

**Authors:** Alexander T. Velaoras, Sabah Iqbal, Alex Silberzweig, Kelly Frasier, Silvija P. Gottesman, Cynthia M. Magro, Sheila Shaigany

**Affiliations:** a Department of Dermatology, Zucker School of Medicine at Hofstra/Northwell, New Hyde Park, New York; b Drexel University College of Medicine, Philadelphia, Pennsylvania; c Rowan-Virtua School of Osteopathic Medicine, East Stratford, New Jersey; d Department of Pathology, Northwell Health, New Hyde Park, New York; e Department of Pathology and Laboratory Medicine, Weill Cornell Medicine, New York, New York

**Keywords:** ADAR1, familial chilblain lupus, IFN-1, interferons, Janus kinase inhibitor, mutation, tofacitinib

## Introduction

Familial chilblain lupus (FCL) is a rare form of monogenic cutaneous lupus presenting in childhood with recurrent, cold-induced, painful violaceous papules at acral sites oftentimes with concomitant inflammatory arthritis, Raynaud’s phenomenon, and cytopenias. As a variant of lupus erythematosus, immunofluorescent testing of lesional skin will show a positive lupus band test. FCL commonly arises from de novo mutations involving interferonopathy-related genes such as 3 prime repair exonuclease 1 (TREX1) and stimulator of interferon genes (STING), subsequently causing overactivated type-I interferon (IFN-I) signaling and autoinflammation.^1^ Adenosine deaminase acting on RNA1 (ADAR1) is another interferonopathy-related gene encoding the enzymatic RNA-editor ADAR1, which functions to suppress innate immune signaling and self-recognition. Pathogenic loss-of-function variants thereby disrupt critical immune-regulation, resulting in overzealous IFN-I upregulation and autoimmunity. While ADAR1 mutation has been reported in monogenic lupus, it is not well described in FCL.^2^ We report a case of pediatric chilblain lupus associated with heterozygous ADAR1 mutation, exhibiting IFN-I overexpression in lesional skin, successfully treated with tofacitinib.

## Case report

A 9-year-old girl with systemic lupus erythematosus (SLE), class V lupus nephritis, and Raynaud’s syndrome presented to dermatology for painful lesions on the hands and feet for several weeks. Family history included a maternal great aunt with SLE and maternal first cousin twice removed with discoid lupus. Diagnosis of SLE was based on positive lupus serologies, cytopenias, hypocomplementemia, proteinuria, and arthritis, which all promptly improved under treatment with mycophenolate mofetil (MMF), hydroxychloroquine, and prednisone (Supplementary Fig 1, available via Mendeley at https://data.mendeley.com/datasets/9nxsg2h584/1). She had been taking MMF for 4 months and tapering prednisone at the time of rash onset.

**Fig 1:**
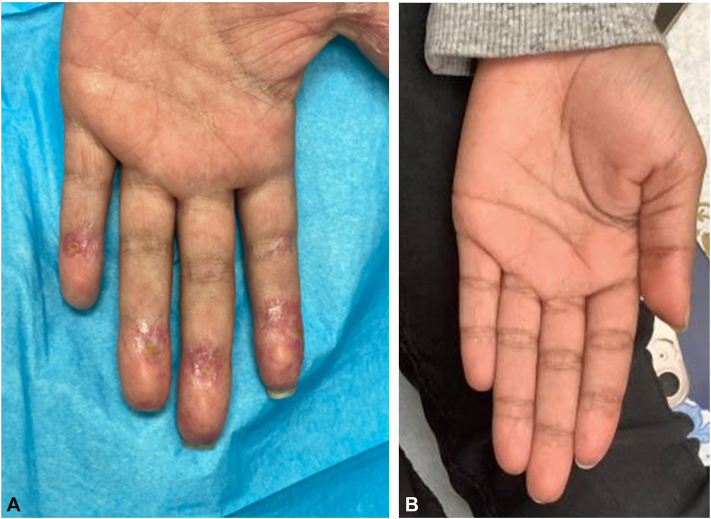
**A,** The patient’s initial presentation with multiple violaceous, telangiectatic papules and erosions on the palmar surfaces of the distal fingertips. **B,** Palmar lesions cleared upon 1-month follow-up after starting tofacitinib.

**Fig 2:**
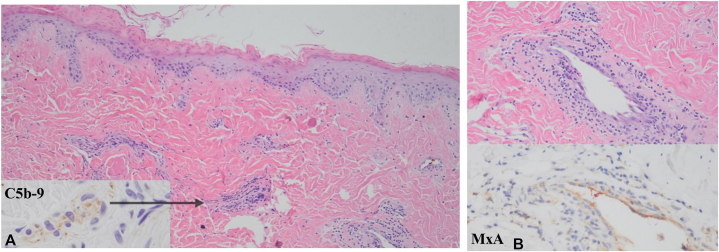
**A,** Punch biopsy of the ventral finger depicts an attenuated epidermis with basement membrane zone thickening and subepidermal vascular dropout and inset immunohistochemical stain with granular endothelial deposition for C5b-9 (hematoxylin-eosin stain, 200× magnification, and IHC inset, 400× magnification). **B,** Shown here is a dilated venule with thickened eosinophilic basement membrane and surrounding lymphocytic infiltrate (hematoxylin-eosin stain, 400× magnification) and MxA immunohistochemical stain surrogate type I interferon marker which highlights staining of endothelial cells and inflammatory cells (IHC, 400× magnification).

Skin examination demonstrated multiple red-purple, eroded, and sclerotic telangiectatic papules on the toes, fingers, palms, and dorsal hands (Fig 1). Punch biopsy demonstrated an attenuated epidermis with a component of interface dermatitis in association with a reticular dermal-based necrotizing lymphocytic vasculitis whereby there was endothelial cell necrosis and denudement, intramural and luminal fibrin deposition (Fig 2, *A*), and basement membrane zone thickening. There were striking microvascular deposits of C5b-9 along with enhanced IFN-I signaling within endothelial cells and inflammatory cells as revealed by the extent of staining for myxovirus resistance protein A (Fig 2, *B*). A diagnosis of chilblains lupus erythematosus was made. Despite escalating doses of prednisone, amlodipine, and intravenous immunoglobulin, her acral lesions persisted (Supplementary Fig 1, available via Mendeley at https://data.mendeley.com/datasets/9nxsg2h584/1). Genetic analysis was pursued using next-generation sequencing and deletion/duplication testing from buccal mucosa, with confirmatory testing through the Pacific Biosciences platform, a test highly orthogonal to next-generation sequencing. Results revealed 2 heterozygous mutations in ADAR1: (1) Pathogenic, loss of function, c.2433_2434del, p.Ala813Glnfs∗29 and (2) Missense, unknown significance, c.1741T>C, p.Ser581Pro. Mutational findings suggested an IFN-1–mediated mechanism, prompting treatment with the Janus kinase inhibitor tofacitinib at 5 mg twice daily. Several weeks later, her rash and pain resolved (Fig 1, *B*), and she began weaning off MMF while continuing intravenous immunoglobulin and hydroxychloroquine. At 8 months of follow-up, she exhibited sustained clinical remission of both rash and nephritis. Serum cytokine testing demonstrated normal levels of inflammatory cytokines including interferon-gamma.

## Discussion

ADAR1 mutation associated with FCL is seldomly reported in existing literature. Loss-of-function mutations in ADAR1, an innate immune regulator responsible for editing dsRNA, can cause Aicardi-Goutieres Syndrome (AGS) through upregulated IFN-I signaling.^3^ While AGS is classically distinguished by infancy-onset encephalopathy and developmental delay, extra-neurologic stigmata including pernio-like lesions and multiorgan involvement often resemble SLE.^3^ This phenotypic overlap likely results from aberrant IFN-I signaling, a well-established driver of pathogenesis in dermatomyositis and SLE, particularly monogenic lupus which exhibits higher IFN-I scores compared to non-Mendelian lupus.^1^

While homozygous and heterozygous mutations of ADAR1 are both associated with AGS, our patient’s single pathogenic mutation has mostly been reported in dyschromatosis symmetrica hereditaria, an autosomal dominant noninflammatory pigmentary disorder, and bilateral striatal necrosis, a disease hallmarked by neuronal death in the striatum, and has been linked with SLE.^2^^,^^4^ Our patient showed no clinical signs of either condition, and diagnostically aligned most with FCL associated with multisystem autoimmunity. Such discordance may be attributed to her additional ADAR1 mutation of unknown significance possibly functioning as a second hit, thereby further reducing enzymatic function and activating IFN-I expression. It remains unclear whether mutations were inherited or occurred de-novo, as the patient’s parents never pursued recommended genetic testing.

Although enhanced IFN-I signaling is a highly characteristic feature of idiopathic and familial perniosis, biopsy findings of a necrotizing thrombogenic lymphocytic vasculitis not limited to the papillary dermis are a hallmark of secondary perniosis associated with systemic disease including SLE and in the perniotic lesions encountered in AGS.^5^^,^^6^ Robust IFN-I expression detected on our patient’s biopsy is a feature of idiopathic perniosis, familial perniosis, childblains lupus, COVID-19 perniosis, and AGS-associated perniosis.^7^ Endothelial cell injury is an intrinsic feature of chilblains lupus. Upregulation of IFN-I signaling is an independent trigger to complement pathway activation whereby C5b-9 is an important effector mechanism of vascular injury and was well exemplified in this case given the extent of microvascular C5b-9.^8^ In addition, IFN-I overexpression also enhances the adaptive humoral immune response, whereby antiendothelial cell antibodies targeting the cutaneous vasculature are also pathogenetically relevant to the microvascular injury seen in chilblain lupus and explain its association with anti-Ro antibodies.^9^

Upregulated IFN-I signaling induces downstream activation of the Janus kinase (JAK)-signal transducer and activator of transcription pathway. Growing evidence points toward JAK inhibitors as a promising treatment, including tofacitinib which demonstrated sustained clinical improvement for up to 24 months in TREX1-associated FCL.^10^ The rationale to treat with anifrolumab is arguably stronger, considering the drug’s mechanistic specificity in targeting the IFN-I receptor and favorable side effect profile compared to JAK inhibitors. Preliminary reports highlight proof-of-concept efficacy for anifrolumab in TREX1-associated FCL, suggesting it may also alleviate syndromic interferonopathies with more severe disease phenotypes.^11^ Despite holding therapeutic promise for FCL, anifrolumab remains underused in this setting, owing to the absence of a Food and Drug Administration–approved pediatric indication. Additional prospective studies investigating safety and efficacy of anifrolumab are warranted to elucidate the role of targeted IFN-1 suppression in individuals harboring interferon-related mutations and assess the utility of genetic testing as a potential treatment guide.

## Conflicts of interest

Dr Shaigany is an advisor for Boehringer Ingelheim, Priovant and Blueprint Medicines. Alexander T. Velaoras, Sabah Iqbal, Alex Silberzweig, Kelly Frasier, Dr Gottesman, and Dr Magro have no conflicts of interest to declare.

## References

[1] Raupov R.K., Suspitsin E.N., Kalashnikova E.M. (2024). IFN-I score and rare genetic variants in children with systemic lupus erythematosus. Biomedicines.

[2] Laxminarayana D., Khan I.U., Kammer G. (2002). Transcript mutations of the alpha regulatory subunit of protein kinase A and up-regulation of the RNA-editing gene transcript in lupus T lymphocytes. Lancet.

[3] Liu A., Ying S. (2023). Aicardi–Goutières syndrome: a monogenic type I interferonopathy. Scand J Immunol.

[4] Kono M., Suganuma M., Dutta A. (2018). Bilateral striatal necrosis and dyschromatosis symmetrica hereditaria: A-I editing efficiency of ADAR1 mutants and phenotype expression. Br J Dermatol.

[5] Kolivras A., Aeby A., Crow Y.J., Rice G.I., Sass U., André J. (2008). Cutaneous histopathological findings of Aicardi-Goutières syndrome, overlap with chilblain lupus. J Cutan Pathol.

[6] Crowson A.N., Magro C.M. (1997). Idiopathic perniosis and its mimics: a clinical and histological study of 38 cases. Hum Pathol.

[7] Magro C.M., Mulvey J.J., Laurence J. (2021). The differing pathophysiologies that underlie COVID-19-associated perniosis and thrombotic retiform purpura: a case series. Br J Dermatol.

[8] Magro C.M., Poe J.C., Kim C. (2011). Degos disease: a C5b-9/interferon-α-mediated endotheliopathy syndrome. Am J Clin Pathol.

[9] Magro C.M., Crowson A.N. (1999). The cutaneous pathology associated with seropositivity for antibodies to SSA (Ro): a clinicopathologic study of 23 adult patients without subacute cutaneous lupus erythematosus. Am J Dermatopathol.

[10] Zhang S., Song J., Yang Y. (2021). Type I interferonopathies with novel compound heterozygous TREX1 mutations in two siblings with different symptoms responded to tofacitinib. Pediatr Rheumatol.

[11] Moran-Alvarez P., Messia V., Matteo V. (2024). Anifrolumab in monogenic lupus caused by TREX1 mutation. J Clin Immunol.

